# Editorial: Neurobiological mechanisms of adjuvant therapies for personalized stroke rehabilitation: towards comprehensive recovery

**DOI:** 10.3389/fnhum.2026.1920955

**Published:** 2026-08-24

**Authors:** Lisa C. Krishnamurthy, Lorie G. Richards, Venkatagiri Krishnamurthy

**Affiliations:** 1 Joseph Maxwell Cleland Atlanta Veterans Affairs (VA) Medical Center, Decatur, GA, United States; 2 Department of Radiology and Imaging Sciences, Emory University, Atlanta, GA, United States; 3 Department of Rehabilitation Medicine, Emory University, Atlanta, GA, United States; 4 Department of Biomedical Engineering, Georgia Tech and Emory University, Atlanta, GA, United States; 5 Department of Occupational and Recreational Therapies, University of Utah, Salt Lake City, UT, United States; 6 Department of Medicine, Division of Geriatrics and Gerontology, Emory University, Atlanta, GA, United States; 7 Department of Neurology, Emory University, Atlanta, GA, United States

**Keywords:** adjuvant therapy, aphasia, biomarkers, cognitive rehabilitation, motor recovery, multimodal therapy, neuromodulation, neuroplasticity

Stroke remains a leading cause of long-term disability worldwide (Feigin et al., 2024). Depending on lesion location, lesion size, stroke subtype, and recovery stage, survivors may experience motor, cognitive, language, sensory, visual, and emotional impairments that limit activity performance, participation, and quality of life (Bernhardt et al., 2017; Boyd et al., 2017). Rehabilitation aims not only to reduce impairment but also to promote meaningful recovery of function through experience-dependent neuroplasticity (Kleim and Jones, 2008; Krakauer, 2006). However, the biological mechanisms that support recovery, the factors that determine treatment response, and the optimal ways to augment rehabilitation remain incompletely understood (Bernhardt et al., 2017; Boyd et al., 2017). This Research Topic brings together studies examining how adjuvant and multimodal therapies may enhance post-stroke recovery by targeting neuroplastic mechanisms, physiological state, neural network function, and patient-specific impairment profiles.

**Figure 1:**
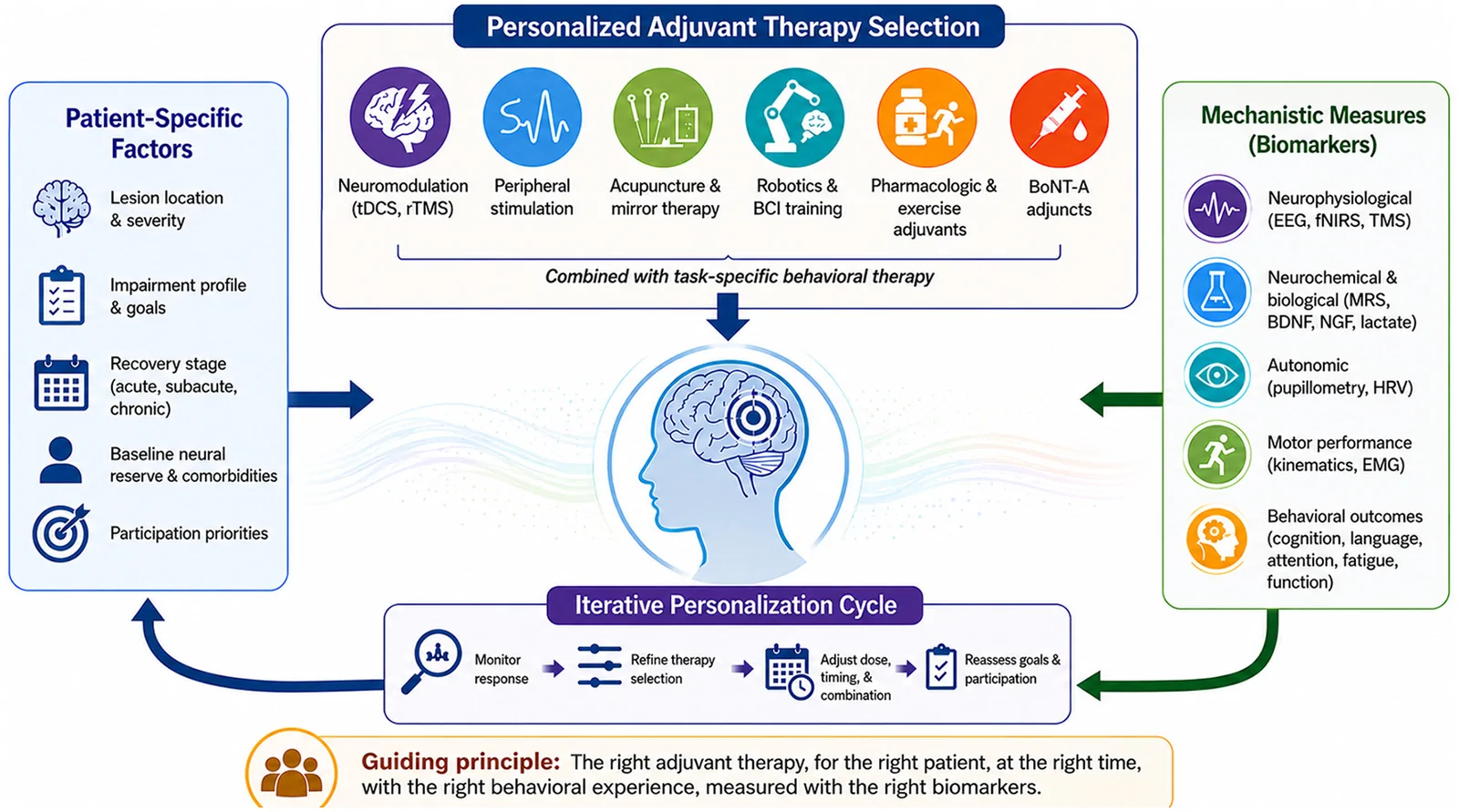
Conceptual model for mechanism-informed adjuvant therapy selection in personalized stroke rehabilitation. Patient-specific factors, including lesion characteristics, impairment profile, recovery stage, baseline neural reserve, comorbidities, and participation priorities, should guide the selection of adjuvant and multimodal therapies. These therapies may include neuromodulation, peripheral stimulation, acupuncture and mirror therapy, robotics and brain-computer interface training, pharmacologic or exercise-based adjuvants, and BoNT-A adjuncts, ideally paired with task-specific behavioral rehabilitation. Mechanistic measures, including neurophysiological, neurochemical, autonomic, motor performance, and behavioral outcomes, can help determine target engagement and guide an iterative personalization cycle in which treatment response is monitored, therapy selection is refined, dose and timing are adjusted, and patient goals are reassessed. The central wave is a visual integration element representing the dynamic interaction among patient-specific factors, therapy selection, mechanistic measurement, and iterative personalization over time. Generation of this figure was assisted by AI. The authors take full responsibility for its content.

A central theme across the articles is that personalized stroke rehabilitation requires moving beyond broad treatment labels toward clearer specification of what an intervention is intended to change, how it is expected to produce that change, and how response should be measured. This aligns with the Rehabilitation Treatment Specification System (RTSS), which emphasizes treatment targets, active ingredients, and hypothesized mechanisms of action (Hart et al., 2019). Within this framework, personalized adjuvant rehabilitation requires determining for whom an intervention works, when it should be delivered, at what dose, with which behavioral therapies, and through which mechanisms (Bernhardt et al., 2017; Boyd et al., 2017; Krakauer, 2006). Figure 1 summarizes this mechanism-informed model, integrating patient-specific factors, adjuvant therapy selection, mechanistic measures, and iterative reassessment.

Several articles in this special topic focus on motor rehabilitation and movement-related impairments after stroke. Reebye et al. provide a conceptual framework for spasticity management by distinguishing adjunctive therapy from multimodal therapy. They emphasize that spasticity and spastic paresis involve neural and non-neural contributors, including muscle overactivity, paresis, impaired motor control, soft-tissue changes, and contracture risk (Reebye et al.). Within this framework, adjunctive therapies are time-specific strategies used to enhance a primary intervention, whereas multimodal therapy refers to a broader, patient-centered program of interventions delivered concurrently or sequentially to address multiple contributors to impairment (Reebye et al.).

Other motor-focused articles illustrate why specifying active ingredients and intended mechanisms matters. Yamamoto et al. report a case study combining repetitive peripheral magnetic stimulation of the elbow and wrist extensors with task-oriented training in a man with chronic severe upper-limb paresis following left putaminal hemorrhage. In this combined intervention, peripheral magnetic stimulation was intended to provide proprioceptive input and modulate muscle activation patterns, while task-oriented training provided repeated, goal-directed motor practice. Despite being 5 years post-stroke, the participant demonstrated clinically meaningful gains in upper-limb motor impairment, improved kinematics, and reduced antagonist co-contraction, although distal voluntary movement and daily arm use remained limited (Yamamoto et al.). Ke et al. extend this theme through a systematic review and meta-analysis of acupuncture combined with mirror therapy for post-stroke limb movement disorders. In this pairing, acupuncture may influence the neurophysiological environment for recovery, while mirror therapy provides visual feedback intended to engage action observation and sensorimotor networks. Across randomized controlled trials, the combined intervention improved motor function, activities of daily living, spasticity, and overall treatment response, while underscoring the need for standardized protocols and objective neurophysiological measures (Ke et al.).

Lu et al. contribute a complementary technology-based approach by examining motor imagery-based brain-computer interface training paired with robotic hand assistance in individuals with ischemic stroke. This intervention links imagined movement, EEG-derived neural signals, robotic movement, and multisensory feedback. Functional gains were observed, and EEG analyses suggested engagement of sensorimotor cortical activity (Lu et al.). However, neural responses varied across individuals, and no clear linear trends emerged across training sessions. These findings reinforce a core message of the Research Topic: neurophysiological responses to rehabilitation are heterogeneous, and biomarkers may need to be interpreted as individualized indicators of cortical engagement rather than universal signatures of recovery.

A second group of articles addresses cognitive, language, attention, and fatigue-related impairments after stroke. Xue et al. describe a randomized controlled trial protocol designed to test whether dual-target anodal transcranial direct current stimulation over the dorsolateral prefrontal cortex and angular gyrus produces greater improvements in post-stroke cognitive impairment than dorsolateral prefrontal stimulation alone. By pairing cognitive outcomes with functional near-infrared spectroscopy measures of cortical activation and connectivity, the protocol offers a mechanism-informed model for evaluating whether broader network targeting may enhance cognitive rehabilitation (Xue et al.).

Li et al. provide clinical evidence for prefrontal neuromodulation in post-stroke cognitive impairment. In their randomized sham-controlled study, repetitive transcranial magnetic stimulation over the left dorsolateral prefrontal cortex was associated with greater cognitive improvement, increased peripheral brain-derived neurotrophic factor and nerve growth factor, and strengthened bilateral prefrontal functional connectivity measured with functional near-infrared spectroscopy (Li et al.). These findings connect behavioral improvement with peripheral neurotrophic markers and network-level physiological change, suggesting that such biomarkers may help clarify mechanisms of response and support treatment stratification.

Harnish et al. broaden the cognitive-language rehabilitation cluster through a narrative review of adjuvant techniques for aphasia rehabilitation, including non-invasive brain stimulation, aerobic exercise, intention treatment, and pharmacotherapies. By describing active ingredients, mechanisms, modulating factors, and efficacy evidence, this review frames aphasia recovery as a neuroplasticity, network, cognitive, and biological-response problem, not only a language-treatment problem (Harnish et al.). Rembrandt and Riley further bridge aphasia, cognition, attention, and fatigue by examining single-session prefrontal transcranial direct current stimulation in people with chronic post-stroke aphasia. Active stimulation did not improve behavioral sustained attention or EEG-derived attention states, but was associated with increased pupil dilation, suggesting locus coeruleus-norepinephrine pathway activation, and appeared to mitigate task-based fatigue relative to sham stimulation (Rembrandt and Riley).

Together, the articles in this Research Topic demonstrate the breadth and complexity of adjuvant therapies in stroke rehabilitation (Reebye et al.; Yamamoto et al.; Ke et al.; Lu et al.; Xue et al.; Li et al.; Harnish et al.; Rembrandt and Riley). Across motor, cognitive, language, attention, fatigue, and spasticity-related impairments, recovery is shaped by interactions among impairment profile, neural target, behavioral task, adjuvant dose, recovery stage, and biological capacity for plasticity. As illustrated in Figure 1, the field is moving toward a model in which adjuvant therapies are selected and combined based on patient-centered goals, mechanistic rationale, and measures of treatment response. Future studies should build on this foundation through mechanism-driven trials that integrate behavioral outcomes with neurophysiological and biological markers (Bernhardt et al., 2017; Boyd et al., 2017), examine dose-response relationships and treatment durability (Bernhardt et al., 2017; Kleim and Jones, 2008; Krakauer, 2006), standardize terminology for adjunctive and multimodal interventions (Reebye et al.), and account for heterogeneity across lesion characteristics, impairment profile, chronicity, and baseline neural reserve (Boyd et al., 2017). Ultimately, advancing personalized stroke rehabilitation will require matching the right adjuvant therapy to the right patient, at the right time, with the right behavioral experience, and with sufficient mechanistic measurement to understand why recovery does—or does not—occur.
